# The Army, the Navy, and Indian Medical Service

**Published:** 1907-09-07

**Authors:** 


### THE ARMY, THE NAVY, AND INDIAN MEDICAL SERVICE.

### The Indian Medical Service.

In a recent number of the *St. Mary's Hospital Gazette*, Captain Brodribb, I.M.S., discusses, in a short and interesting article, which the intending candidate would do well to read, the pros and cons of the service. "For the poor man," he decides, "the service is a great investment. By the expenditure of £100 on uniform (which can be paid after entering the service, if need be) he is able to secure an income which, taking the pension into consideration, cannot be put at less than that of a practice of £800 to £1,000 a year—that is supposing he never does anything great in the service. . . . Life for a single man in cantonments, with a pony, mess, and club bill, costs about 350 to 400 rupees per month, while leave costs anything you like to spend on it. . . Married life is, of course, much more expensive. . . .

"The life at first is often a very lonely one, with perhaps one or two other white men in an enormous district; but the junior man in civil generally draws more than the man of the same service in military, and of course there are stations for the more senior ranks which, if he gets them, bring in a large income, though these are few and far between, and the work extraordinarily hard and wearying in this climate. Still, a man must not expect to get Rx.200 to Rx.1,000 a month for doing nothing. There are other branches, as under the Foreign Office; working in Native States; gaols; essay; botanical, research, military administrative posts; staff of medical colleges all with their advantages and disadvantages. Nowadays it takes about three years (for gaols) to eight years (for the best provinces) to get out of the military into these branches, but you may, up to three years after leaving it, elect to return to your own regiment if you are *pukka* with the regiment; so it is well to get *pukka* with your regiment before leaving it, otherwise you would have to return to the bottom of the officiating list. To get *pukka* (that is, actually *appointed*, and not *attached*) in the regiment will take four to five years.

"Now let us see what is to be said on the other side, that is, against the service. Most important, perhaps, is the country itself; it is not a white man's country, and as long as you stay you never get accustomed to the fierce heat of the Indian summer. There are few things more deadly than a series of really hot Indian nights, when sleep becomes out of the question, and one gets up in the morning weary in body and soul. The new arrival is the happiest, enjoying many things that he did not get at home; lengthy if bad dinners; lots of servants; authority out of all proportion to what he had at home; more means and less work; during the middle of his service he yearns for home; at the end of it he has become separated from home ties; has lost energy and interest, and often becomes a time-server unfitted for any other walk in life.

"Influence plays a great part in a man's career in India; without it, it is likely that he will never get a chance."

He sums up by declaring: "I think it is the best service in the world, comparing favourably with private practice in England. It offers no real chance of failure, has few responsibilities, many and varied branches to choose from, a certain living to death, and, if married, a competence which can be increased by insurance for those that remain behind."

Candidates must be natural-born subjects of His Majesty between 21 and 28 years of age at the date of the examination, of sound bodily health, and in the opinion of the Secretary of State for India in Council in all respects suitable to hold commissions in the Indian Medical Service. They may be married or unmarried. They must possess, under the Medical Acts in force at the time of their appointment, a registrable qualification to practise both medicine and surgery in great Britain and Ireland.

After passing examination, the successful candidates will be required to attend one entire course of practical instruction at the Army Medical School and elsewhere, as may be decided, in :

(1) Hygiene; (2) Military and Tropical Medicine; (3) Military Surgery; (4) Pathology of diseases and injuries incidental to Military and Tropical Service.

This course will be of not less than four months' duration.

### The Navy.

This service is only open to the graduate of European birth between twenty-one and twenty-eight years of age. He must pass an entrance examination and gain at least one-third of the possible marks. Successful candidates immediately after passing the examination will receive commissions as surgeons in the Royal Navy, and undergo a course of practical instruction in naval hygiene, the diseases of warm climates, bacteriology, surgery of gunshot wounds, etc., radiography, and the photography in connection with it at Haslar Hospital. Surgeons are only required to provide themselves with a regulation case of pocket instruments, a stethoscope, and three clinical thermometers. All other instruments are provided at the public expense.

Every medical officer will be required to undergo a post-graduate course of three months' duration at a metropolitan hospital once in every eight years (should the exigencies of the service permit), and this as far as possible during his surgeon's, staff surgeon's, and fleet surgeon's period of service. While carrying out this course the medical officer will be borne on a ship's books for full pay, and will be granted lodging and provision allowances, and travelling expenses as for service under the regulations to and from his home or port; the fees for each course (not exceeding £25) will be paid by the Admiralty on the production of vouchers at the end of the course. The medical officer will be required to produce separate certificates of efficient attendance in the following : I. The medical and surgical practice of the hospital; II. A course of operative surgery on the dead body; III. A course of bacteriology; IV. A course of ophthalmic surgery, particular attention being paid to the diagnosis of errors of refraction; V. A practical course of skiagraphy.

### The Army.

A candidate for a commission in the Royal Army Medical Corps must be 21 years and not over 28 years of age at the date of the commencement of the entrance examination. He must possess, under the Medical Acts in force in the United Kingdom at the time of his appointment, a registered qualification to practise. He must pass an entrance examination, and, having gained a place, must undergo two months' instruction in hygiene and bacteriology, and afterwards proceed to Aldershot for instruction in technical duties of the corps.

